# The Role of Gait Characteristics in Understanding Aging in Midlife

**DOI:** 10.1093/geroni/igaf122.960

**Published:** 2025-12-31

**Authors:** Roy Tzemah-Shahar, Merav Asher, Maayan Agmon

**Affiliations:** University of Haifa, Haifa, HaZafon, Israel; University of Haifa, Haifa, HaZafon, Israel; University of Haifa, Haifa, HaZafon, Israel

## Abstract

Gait quality is a hallmark of aging, with its decline signalling cognitive deterioration, fall risk, and mortality. Gait has been linked to Biological Age (BA), a quantitative measure of aging, estimated by physiological or behavioral markers such as Physical Capacity (PC). However, most studies of BA focus on older adults, leaving midlife largely unexplored. This study examines the relationship between gait characteristics and BA in midlife. We conducted a cross-sectional study of healthy midlife adults (N = 95, mean age 45.6±0.7, 48% women). BA was estimated using the Klemera-Doubal method with physiological biomarkers and a PC battery assessing muscular strength, endurance, flexibility, balance, and agility. Gait speed and variability were evaluated under single- and dual-task walking conditions. PC composite scores were negatively associated with BA when controlling for age, education, and smoking (adjusted R²=0.19; b=-0.42, p < 0.001), with endurance, flexibility, and balance remaining significant predictors. Gait speed and stride length variability were not significantly associated with BA (Pearson’s r = 0.17-0.01, p = 0.10-0.99). However, gait speed in both single- and dual-task conditions was significantly correlated with PC scores (Pearson’s r = 0.61, p < 0.001 and r = 0.51, p < 0.001, respectively). While gait is a key aging marker in older adults, these findings suggest that it may not fully capture physiological aging in midlife. A comprehensive PC assessment may better detect subtle aging-related changes, offering a valuable tool for early interventions to promote healthy aging.

